# Flower Color and Seed Coat Color as a Phenotypic Marker: Correlations with Fatty Acid Composition, Antioxidant Properties, and Metabolite Profiles in Safflower (*Carthamus tinctorius* L.)

**DOI:** 10.3390/ijms26073105

**Published:** 2025-03-27

**Authors:** Weilan Li, Eun-Gyeong Kim, Dongho Lee, Young-Min Choi, Jae-Eun Lee, Sookyeong Lee, Gi-An Lee, Eunae Yoo

**Affiliations:** 1 National Agrobiodiversity Center, National Institute of Agricultural Sciences, Rural Development Administration, Jeonju 54874, Republic of Korea; athancol@163.com (W.L.); keg950@korea.kr (E.-G.K.); jnlee88@korea.kr (J.-E.L.); xsanta7@korea.kr (S.L.); 2 Department of Plant Biotechnology, College of Life Sciences and Biotechnology, Korea University, Seoul 02841, Republic of Korea; dongholee@korea.ac.kr; 3 National Herb Experiment Station, Medicinal Herb Resource Research Institute, Jeollabuk-Do Agricultural Research & Extension Services, Namwon 55720, Republic of Korea; cym9288@korea.kr

**Keywords:** flower color, safflower, phenotypic diversity, metabolite profiles, trait-based breeding

## Abstract

Safflower (*Carthamus tinctorius* L.) is a versatile oilseed crop valued for its adaptability, high oil quality, and antioxidant properties. This study investigates the influence of flower color (FC) on the phenotypic diversity of 172 safflower accessions, analyzing agronomic traits, metabolite profiles, and antioxidant capacities. Frequency distribution, effect size, principal component analysis (PCA), and network analysis were employed to elucidate trait associations and interrelationships. FC significantly impacted traits such as oleic acid (OA), linoleic acid (LA), oleic desaturation ratio (ODR), and N-feruloylserotonin (FS), with large effect sizes (η^2^ > 0.16). Medium effects were observed for 2,2′-azino-bis(3-ethylbenzothiazoline-6-sulfonic acid) (ABTS) scavenging capacity, palmitic acid (PA), and flowering date (FD). PCA and network analyses highlighted relationships between FC and other fatty acid and antioxidant traits. Qualitative traits such as seed coat color (SCC) and thorn of involucre (TI) also showed significant associations with FC, underscoring its role as a phenotypic marker. These findings provide a robust framework for trait-based breeding strategies in safflower and emphasize the need for further genetic validation of these associations.

## 1. Introduction

Safflower (*Carthamus tinctorius* L.) is an important oilseed crop known for its adaptability to diverse environmental conditions and its versatility in agricultural and industrial applications [1]. Historically cultivated for its vibrant floral pigments, safflower has recently garnered increased attention due to its oil composition, which is rich in unsaturated fatty acids, particularly oleic and linoleic acids [2]. Additionally, both safflower seeds and flowers exhibit notable antioxidant properties, enhancing their value in nutritional and pharmaceutical contexts [3]. The phenotypic diversity of safflower, encompassing both quantitative and qualitative traits, provides a critical foundation for breeding programs aimed at improving oil yield, antioxidant capacity, and morphological characteristics.

Among these phenotypic traits, flower color (FC) has been recognized not only as a visually distinguishable characteristic but also as a potential phenotypic marker associated with various agronomic and biochemical traits [4]. Previous studies have suggested that FC may influence seed coat color (SCC), thorn of involucre (TI), and other traits; however, comprehensive analyses quantifying the impact of FC on both quantitative and qualitative traits remain limited. Investigating these associations offers valuable insights into the complex genetic and physiological interactions underlying safflower phenotypes.

In addition to its agronomic relevance, the analysis of safflower’s metabolite composition—particularly its fatty acid profiles—offers promising avenues for improving oil quality [5]. Oleic acid (OA), linoleic acid (LA), and alpha-linolenic acid (ALA) are key components that determine the nutritional and industrial value of safflower oil. Moreover, antioxidant capacities, as measured by total phenolic content (TPC), 2,2′-azino-bis(3-ethylbenzothiazoline-6-sulfonic acid) (ABTS) radical scavenging capacity, and 2,2-diphenyl-1-picrylhydrazyl (DPPH) radical scavenging capacity, are crucial for enhancing the health benefits and shelf life of safflower-derived products. Understanding the interactions between FC and these traits is essential for developing targeted breeding strategies [6].

In addition to their oil content, safflower seeds are rich in phenolic compounds, which are well known for their antioxidant properties and potential medicinal applications. Polyphenols, extensively documented for their broad spectrum of biological activities, exhibit anti-inflammatory, anticancer, antiallergic, and cardioprotective effects [7,8,9]. These compounds function as natural antioxidants, neutralizing harmful reactive oxygen species and thereby contributing to overall health [10]. Among the bioactive compounds found in safflower seeds, *N*-(*p*-coumaroyl)serotonin (CS) and *N*-feruloylserotonin (FS) are the primary serotonin derivatives; both reported to be abundant in safflower seeds. CS and FS are particularly noteworthy due to their potent bioactivity.

Network analysis and multivariate techniques, such as principal component analysis (PCA), provide robust tools for unraveling complex trait relationships in crops. Integrating these approaches, this study aims to: (1) analyze the frequency distribution of agronomic traits, metabolite contents, and antioxidant capacities in 172 safflower accessions; (2) assess the impact of flower color (FC) on quantitative traits using effect size (Eta-squared, η^2^) and statistical significance (*p*-value); (3) explore trait relationships influenced by FC through PCA and correlation network analysis; and (4) investigate the associations between FC and qualitative traits (SCC and TI) using network visualization. This comprehensive analysis will enhance our understanding of the role of FC in shaping safflower phenotypic diversity and its implications for trait-based breeding strategies.

## 2. Results

### 2.1. Agriculture Traits, Metabolite Content, Antioxidant Capacities Frequency Distribution in Safflower

**Figure 1:**
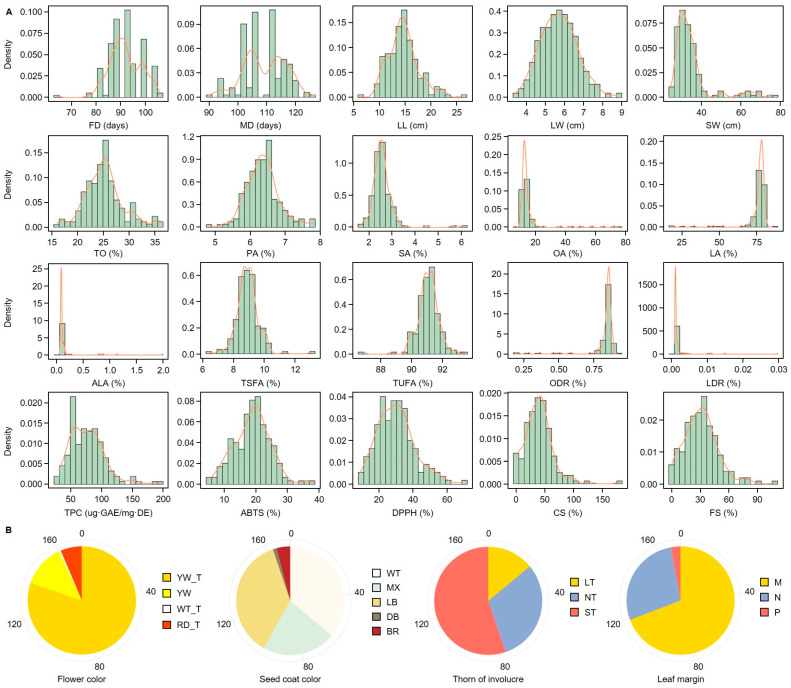
Distribution of quantitative traits and categorical characteristics in safflower accessions. (**A**) Density plots showing the distribution of quantitative traits, including flowering date (FD), maturing date (MD), leaf length (LL), leaf width (LW), 1000-seed weight (SW), total oil content (TO), palmitic acid (PA), stearic acid (SA), oleic acid (OA), linoleic acid (LA), alpha-linolenic acid (ALA), total saturated fatty acids (TSFA), total unsaturated fatty acids (TUFA), oleic de-saturation ratio (ODR), linoleic de-saturation ratio (LDR), total phenolic content (TPC), ABTS and DPPH assays, *N*-(*p*-coumaroyl)serotonin (CS); *N*-feruloylserotonin (FS). (**B**) Pie charts representing the proportions of categorical traits, including flower color (yellow, yellow_T, white_T, and red_T), seed coat color (white, mixed, light brown, dark brown, and brown), thorn presence on involucre (long thorn, no thorn, and short thorn), and leaf margin characteristics (many, no, or present).

The frequency distribution of 172 safflower accessions (Table S1) was analyzed for agronomic traits, metabolite contents, and antioxidant capacities (Figure 1A). For clarity, the abbreviations used for agronomic and biochemical traits throughout this study are listed in Table 1. The flowering date (FD) varied widely among accessions, with a mean of 91.9 ± 6.55 days, while the maturing date (MD) averaged 109.40 ± 7.31 days. Leaf length (LL) and leaf width (LW) exhibited mean values of 14.64 ± 3.05 cm and 5.70 ± 0.95 cm, respectively, both following a normal distribution pattern. The 1000-seed weight (SW) also showed considerable variation, with an average of 34.74 ± 9.71 g.

For fatty acid components, total oil (TO), palmitic acid (PA), and stearic acid (SA) averaged 25.23 ± 3.78%, 6.35 ± 0.47%, and 2.59 ± 0.50%, respectively, and exhibited distributions similar to a normal curve (Figure 1A and Figure S1). Conversely, oleic acid (OA), linoleic acid (LA), and alpha-linolenic acid (ALA) showed skewed distributions, with mean values of 15.27 ± 9.45%, 75.67 ± 9.34%, and 0.12 ± 0.19%, respectively. Total saturated fatty acid (TSFA) and total unsaturated fatty acid (TUFA) averaged 8.94 ± 0.74% and 91.06 ± 0.74%, respectively, and followed near-normal distributions. The oleic desaturation ratio (ODR) and linoleic desaturation ratio (LDR) were also skewed, with average values of 0.83 ± 0.10% and 0.002 ± 0.003%, respectively.

Among the antioxidant capacity measures, total phenolic content (TPC), ABTS, and DPPH exhibited normal distribution patterns, with averages of 76.43 ± 28.46 µg·GAE/mg·DE, 18.94 ± 5.74%, and 30.16 ± 10.94%, respectively. The serotonin derivatives, CS and FS, showed diverse distribution patterns, with mean values of 39.94 ± 24.49% and 31.31 ± 17.93%, respectively.

For qualitative traits (Figure 1B), accessions with yellow-transition (YE_T) flowers were the most frequent, followed by those with yellow (YE), white (WT), and red-transition (RD_T) colors. In terms of SCC, light brown (LB) was the most prevalent, followed by white (WT), mixed (MX), brown (BR), and dark brown (DB). Regarding the TI, accessions with short thorns (ST) were most common, followed by those with no thorns (NT) and long thorns (LT). For leaf margin (LM), the “many” type (M) was most frequent, while the “present” type (P) was the least observed.

### 2.2. The Effect of FC on Agronomic Traits, Metabolite Content, and Antioxidant Capacities

The impact of FC and SCC on various quantitative traits—including agronomic traits, metabolite contents, and antioxidant capacities—was assessed using effect size (η^2^) and statistical significance (*p*-value) (Table 2). Among the traits influenced by FC, OA (η^2^ = 0.22, *p* < 0.001), LA (η^2^ = 0.22, *p* < 0.001), ODR (η^2^ = 0.22, *p* < 0.001), and FS (η^2^ = 0.17, *p* < 0.001) exhibited large effect sizes, indicating significant influences. Medium effect sizes were observed for ABTS (η^2^ = 0.11, *p* < 0.001), PA (η^2^ = 0.07, *p* = 0.009) and FD (η^2^ = 0.06, *p* = 0.016). LW showed statistical significance (η^2^ = 0.05, *p* = 0.038), but with only a small effect size. Other traits—including MD, LL, SW, TO, SA, ALA, TSFA, TUFA, LDR, TPC, DPPH, and CS—exhibited neither statistical significance nor meaningful effect sizes.

For SCC, large effect sizes were observed for ABTS (η^2^ = 0.24, *p* < 0.001), TO (η^2^ = 0.18, *p* < 0.001), and PA (η^2^ = 0.16, *p* < 0.001), suggesting strong influences. Medium effect sizes were found for FS (η^2^ = 0.11, *p* < 0.01), CS (η^2^ = 0.09, *p* < 0.01), and DPPH (η^2^ = 0.08, *p* < 0.05), indicating moderate associations with SCC. In contrast, traits such as FD, MD, LL, LW, SW, SA, OA, LA, ALA, TSFA, TUFA, ODR, LDR, and TPC did not show significant or meaningful effect sizes in relation to SCC.

**Figure 2:**
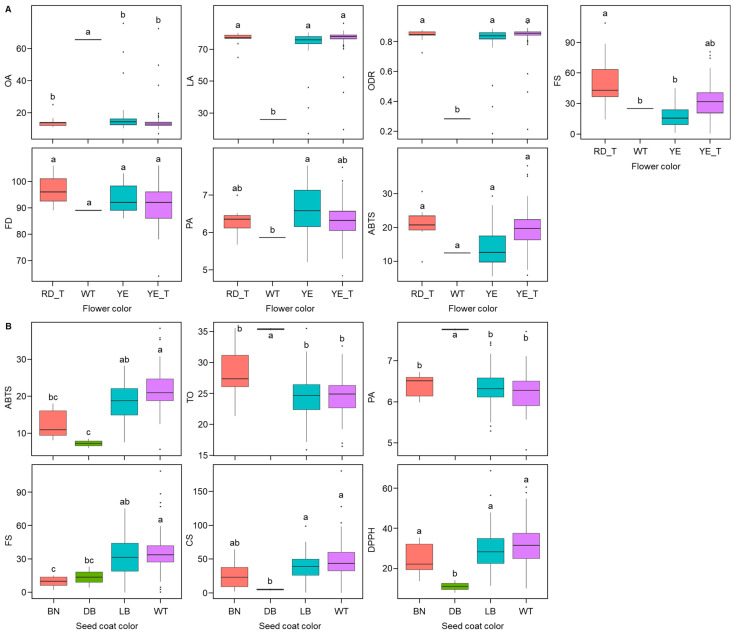
Box plots showing the variation in quantitative traits across different flower color (FC) and seed coat color (SCC) types in safflower. (**A**) The traits analyzed include oleic acid (OA), linoleic acid (LA), oleic de-saturation ratio (ODR), *N*-feruloylserotonin (FS), flowering date (FD), palmitic acid (PA), and ABTS. FC types are categorized as Red_T (RD_T), White (WT), Yellow (YE), and Yellow_T (YE_T). (**B**) The traits analyzed include 2,2′-azino-bis(3-ethylbenzothiazoline-6-sulfonic acid) (ABTS), total oil content (TO), palmitic acid (PA), *N*-feruloylserotonin (FS), *N*-(*p*-coumaroyl)serotonin (CS), and 2,2-Diphenyl-1-picrylhydrazyl (DPPH). SCC types are categorized as brown (BN), dark brown (DB), light brown (LB), and white (WT). Different letters (a, b, c) above the boxes indicate statistically significant differences among groups (*p* < 0.05) as determined by Duncan’s multiple range test.

For traits with statistically significant differences and medium to large effect sizes, box plots were used to compare mean values across FC and SCC categories (Figure 2). OA content was higher in accessions with WT flowers compared to those with RD_T, YE, and YE_T flowers. In contrast, LA and ODR levels were higher in accessions with RD_T, YE, and YE_T flowers than in those with WT flowers. FS content was greater in accessions with RD_T flowers than in those with WT and YE flowers. PA content was higher in YE-flowered accessions than in WT-flowered ones. Although FC had a significant impact on FD and ABTS, no significant differences were observed among flower color categories for these traits.

In Figure 2B, the distribution of safflower accessions across SCC categories is shown using box plots. Accessions with WT seed coat color exhibited higher ABTS levels than those with BN and DB colors. Conversely, DB-colored accessions showed higher TO and PA contents compared to those with BN, LB, and WT colors. FS content was greater in WT accessions than in those with BN and DB colors. CS levels were higher in LB and WT accessions than in DB ones. Lastly, DPPH activity was greater in BN, LB, and WT accessions compared to DB accessions.

### 2.3. PCA and Network Analysis by FC and SCC

**Figure 3:**
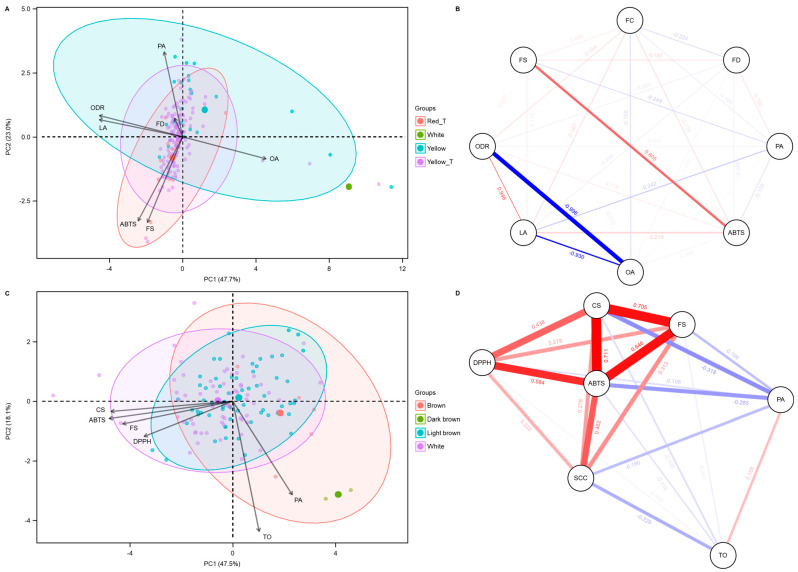
Principal component analysis (PCA) and correlation network of quantitative traits in safflower accessions grouped by flower color (FC) and seed coat color (SCC) types. (**A**) PCA biplot showing the distribution of safflower accessions based on quantitative traits, with the first principal component (PC1, 47.7%) and the second principal component (PC2, 23.0%) explaining the variance. Vectors represent the contributions of key traits, including oleic acid (OA), linoleic acid (LA), palmitic acid (PA), oleic desaturation ratio (ODR), 2,2′-azino-bis(3-ethylbenzothiazoline-6-sulfonic acid) (ABTS), flowering date (FD), and *N*-feruloylserotonin (FS). Groups are differentiated by flower color types: Red_T (RD_T), White (WT), Yellow (YE), and Yellow_T (YE_T). (**B**) Correlation network depicting the relationships between quantitative traits. Edges represent correlations, with thickness and color indicating the strength and direction of the correlation (red for positive, blue for negative). Notable correlations include strong positive correlations between ODR and LA, and a strong negative correlation between ODR and OA. This network highlights the interconnectedness of their influence on FC in safflower. (**C**) PCA biplot based on SCC. The PCA biplot reveals the distribution of safflower accessions according to SCC, with PC1 (47.5%) and PC2 (23.1%) explaining the variance. The vectors indicate the contributions of traits such as ABTS, *N*-(*p*-coumaroyl)serotonin (CS), FS, 2,2-Diphenyl-1-picrylhydrazyl (DPPH), PA, and total oil content (TO), with different seed coat color groups—brown (BN), dark brown (DB), light brown (LB), and WT—showing distinct clustering patterns. (**D**) Correlation network based on SCC. Notable patterns include strong positive correlations between CS and ABTS, and between ABTS and FS, as well as significant negative correlations between SCC and PA and TO.

PCA and network analyses were conducted on traits significantly influenced by FC to explore their relationships and variation patterns (Figure 3). In the FC analysis, the first two principal components (PC1 and PC2) together explained 70.7% of the total trait variation, with PC1 and PC2 accounting for 47.7% and 23.0% of the variance, respectively (Figure 3A). In the PCA biplot, OA was positioned in the opposite direction to other traits along the PC1 axis, indicating a strong negative relationship between OA and the other variables. Similarly, along the PC2 axis, FD, PA, ODR, and LA were located opposite to OA, ABTS, and FS, suggesting a negative association between these two groups of traits. The biplot also revealed distinct clustering of FC groups (RD_T, WT, YE, and YE_T).

In the SCC-based PCA (Figure 3C), a distinct variation pattern was observed for traits such as TO, PA, DPPH, ABTS, CS, and FS across different SCC groups (BN, DB, LB, and WT). PC1 and PC2 accounted for 47.5% and 18.1% of the total variation, respectively. The biplot showed that traits were distributed differently among SCC groups, with the BN group predominantly clustered along the positive PC1 axis, while the LB and WT groups were more dispersed. TO and PA were strongly associated with the BN group, as indicated by their positions on the positive side of PC1. In contrast, CS, ABTS, FS, and DPPH were more closely associated with the WT group, aligning along the negative side of both PC1 and PC2.

The correlation network analysis revealed significant associations between FC and several quantitative traits, including FD, PA, ABTS, OA, LA, ODR, and FS (Figure 3B). FC showed the strongest negative correlation with FD (r = −0.224), followed by OA (r = −0.158) and PA (r = −0.109). In contrast, positive correlations were observed between FC and ABTS (r = 0.175), LA (r = 0.167), ODR (r = 0.164), and FS (r = 0.100). Moreover, a strong positive correlation was identified between ABTS and FS (r = 0.605), indicating that higher FS levels are associated with increased ABTS activity. Conversely, a nearly perfect negative correlation was observed between OA and ODR (r = −0.998), suggesting that as OA increases, ODR decreases sharply.

The correlation network between SCC and quantitative traits, including TO, PA, ABTS, DPPH, CS, and FS, was also examined (Figure 3D). SCC exhibited moderate to strong positive correlations with DPPH (r = 0.222), CS (r = 0.276), FS (r = 0.313), and ABTS (r = 0.462). In contrast, negative correlations were observed between SCC and TO (r = −0.229) and PA (r = −0.190), indicating an inverse relationship.

### 2.4. Network Analysis Between FC and Qualitative Traits

**Figure 4:**
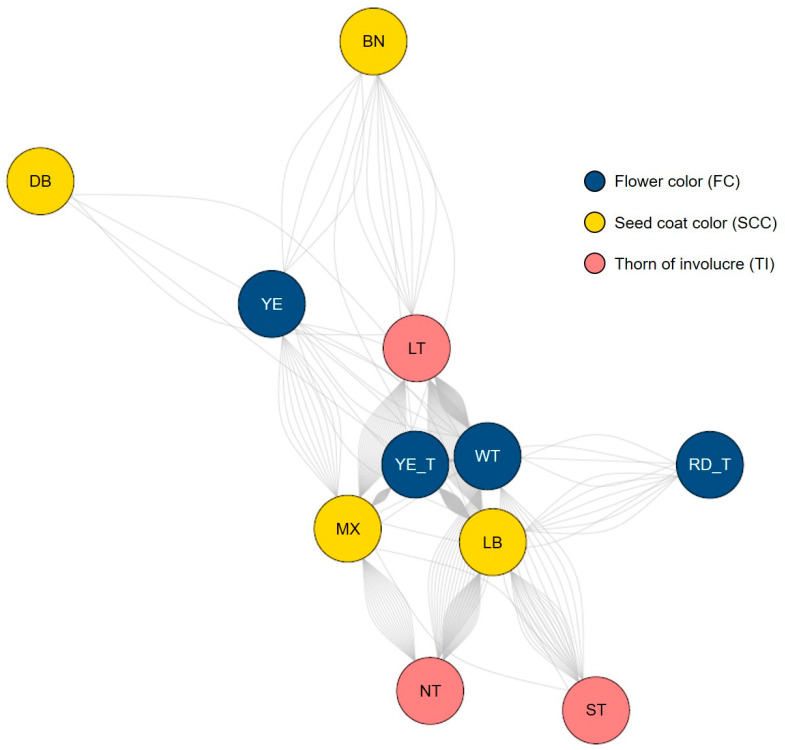
The network represents the relationships between three major trait categories: flower color (navy nodes), seed coat color (yellow nodes), and thorn of involucre (red nodes). Edges indicate connections based on accessions. Notable clusters include flower color traits such as yellow (YE) and yellow transition (YE_T), and WT, seed coat colors such as light brown (LB) and mixed (MX), and thorn traits including long thorn (LT), no thorn (NT), and short thorn (ST). The visualization highlights interactions and co-occurrences among the categorical traits within the safflower germplasm.

Network analysis revealed significant associations between FC and qualitative traits, including SCC and TI (Table 3 and Figure 4). Chi-square analysis confirmed these associations, with *p*-values of 0.005 for SCC and 0.001 for TI (Table 3). The corresponding Cramer’s V values indicated a moderate relationship for SCC (0.235) and a moderately strong relationship for TI (0.260). In contrast, the association with LM was not statistically significant (*p* = 0.342), showing a very weak relationship (Cramer’s V = 0.140). These findings highlight varying levels of correlation between FC and qualitative traits in safflower.

The network visualization further illustrates the strength and nature of these relationships, emphasizing distinct clusters and trait interconnections (Figure 4). In this network, nodes with higher edge density are identified as central hubs, and shorter edge lengths indicate stronger associations. Based on the analysis, nodes representing YE_T and WT (FC), LT (TI), and MX and LB (SCC) were identified as key hubs. Specifically, YE_T (FC) was strongly associated with LB and MX (SCC), while WT (FC) showed a strong connection with LT (TI).

## 3. Discussion

This study provides a comprehensive evaluation of the relationships between FC and both quantitative and qualitative traits in safflower. By integrating frequency distribution analysis, effect size evaluation, PCA, and network analysis, we identified significant associations that underscore the phenotypic diversity of safflower and its potential for trait-based selection in breeding programs.

### 3.1. Impact of FC on Quantitative Traits

The results demonstrated that FC significantly influences several quantitative traits, including OA, LA, ODR, and FS, which exhibited large effect sizes (η^2^ > 0.16). Biologically, the impact of FC on fatty acid profiles and antioxidant capacities suggests that flower pigmentation may be linked to underlying metabolic pathways that regulate oil composition. For instance, accessions with white flowers (WT) exhibited higher oleic acid levels—known for their stability and health benefits—whereas red-flowered (RD_T) accessions showed higher antioxidant contents, particularly N-feruloylserotonin (FS). This suggests that the biosynthetic pathways responsible for flower pigmentation may share common metabolic intermediates with those involved in fatty acid biosynthesis and antioxidant production [11].

Furthermore, the strong effects observed between FC and seed oil components such as OA, LA, and ODR indicate that FC could be utilized as an early selection criterion in breeding programs aimed at improving specific oil profiles in safflower cultivars. The observed relationship between FC and fatty acid composition also supports the hypothesis that pigment biosynthesis may be closely associated with seed composition, thereby reinforcing the potential of colour-based selection in safflower improvement.

Medium effect sizes were found for ABTS, PA, and FD, indicating a moderate influence of FC on these traits. However, the lack of significant differences in FD across FC categories suggests that flower colour may have a limited effect on flowering time compared to its influence on biochemical traits [12]. Notably, traits such as ALA and TPC exhibited neither statistical significance nor meaningful effect sizes, implying that their variation may be controlled by factors independent of FC.

### 3.2. PCA and Trait Interactions

PCA revealed that the first two principal components (PC1 and PC2) explained 70.7% of the total variation among traits, providing a clear visualization of trait relationships influenced by FC. OA was positioned in the opposite direction to LA, ODR, and FS along PC1, indicating negative associations. Similarly, along PC2, OA, ABTS, and FS were oriented opposite to FD, PA, ODR, and LA, highlighting distinct groupings of inversely related traits. The clustering of FC groups—Red_T, White, Yellow, and Yellow_T—underscores the phenotypic differentiation driven by flower colour, particularly in traits related to fatty acid composition and antioxidant capacity [13].

### 3.3. Network Analysis of Quantitative Traits

Correlation network analysis further substantiated the relationships observed in the PCA. FC exhibited the strongest negative correlation with FD (r = −0.224), followed by OA (r = −0.158), suggesting that accessions with certain flower colours may be predisposed to specific flowering times and oil profiles. Positive correlations were observed between FC and ABTS (r = 0.175), LA (r = 0.167), ODR (r = 0.164), and FS (r = 0.100), implying that FC may be associated with enhanced antioxidant capacities and modified oil composition. The strong positive correlation between ABTS and FS (r = 0.605) highlights a potential linkage in antioxidant properties, while the near-perfect negative correlation between OA and ODR (r = −0.998) suggests a metabolic trade-off in fatty acid biosynthesis.

### 3.4. Associations Between FC and Qualitative Traits

The network analysis of qualitative traits revealed significant associations between FC, SCC, and TI. Nodes representing YE_T and WT in the FC category, LT in the TI category, and MX and LB in the SCC category emerged as central hubs, indicating their pivotal roles in the network. Specifically, YE_T was strongly associated with SCC categories LB and MX, while WT showed a strong connection with LT in the TI category. These findings suggest that flower colour may serve as a phenotypic marker for predicting seed coat colour and thorn morphology—traits that are critical for safflower’s adaptability and agronomic utility. The observed clustering and edge densities emphasize the complex interplay between FC and qualitative traits, underscoring the potential of network-based approaches to identify key trait combinations for targeted breeding [14].

While our study focused on seed-derived phenolic traits, future research incorporating flavonoid profiling in floral tissues will be essential to more fully elucidate the metabolic linkages between flower pigmentation and biochemical seed traits. In conclusion, this study highlights the multifaceted role of FC in shaping phenotypic diversity in safflower. By integrating advanced statistical and network-based approaches, it provides a comprehensive framework for understanding trait interactions and identifying phenotypic markers for breeding. Future research should focus on validating these associations through genetic and molecular analyses to further unravel the mechanisms underlying the observed phenotypic relationships.

## 4. Materials and Methods

### 4.1. Reagents and Chemicals

Folin–Ciocalteu phenol reagent, DPPH, ABTS, potassium persulfate, 14% boron trifluoride-methanol (BF_3_-methanol), anhydrous sodium sulfate, *n*-hexane, sodium hydroxide, and fatty acid standards—including palmitic (16:0, PA), stearic (18:0, SA), oleic (18:1, OA), linoleic (18:2, LA), and linolenic (18:3, ALA) acids—were procured from Sigma-Aldrich (St. Louis, MO, USA). Specifically, their methyl esters forms were used: methyl palmitate (P5177-1G), methyl stearate (S5376-1G), methyl oleate (311111-5G), methyl linoleate (L1876-1G), and methyl linolenate (L2626-100MG). Chloroform, ethanol, and methanol were also obtained from the same supplier. *N*-(*p*-coumaroyl)serotonin (CS, CFN9112-5mg) and *N*-feruloylserotonin (FS, sc-498142-25mg) were purchased from Santa Cruz Biotechnology (Santa Cruz, CA, USA). All chemicals used were of analytical grade and applied without further purification.

### 4.2. Safflower Cultivation and Sample Preparation

A total of 172 safflower accessions from Africa (*n* = 2), Asia (*n* = 111), Europe (*n* = 9), and North America (*n* = 50) were obtained from the gene bank of the National Agrobiodiversity Center, Rural Development Administration, Jeonju, South Korea. Seeds were sown on 31 March 2021 and cultivated under uniform field conditions at the Herb Experiment Station, Medicinal Herb Resource Research Institute, Jeollabuk-Do Agricultural Research and Extension Services, Namwon, South Korea. The plants were directly sown in the field with 15 cm spacing between individuals. Key agro-morphological traits, including FC transition (Figure S2) and SCC, were closely monitored throughout the growth period. At full maturity, seeds were harvested and dried in a VS-1202D drying oven (Vision Scientific, Bucheon, Korea) at 45 °C for three days. The 1000-seed dry weight was measured. Dried samples were ground, passed through a 315 µm sieve, and stored at −20 °C until analysis.

### 4.3. Safflower Agronomic Traits Investigation

Agronomic traits were evaluated during cultivation, focusing on both quantitative and qualitative characteristics. Quantitative traits included FD, defined as the number of days from sowing to flowering; MD, the number of days from sowing to harvest; LL, measured as the longest vertical axis of the leaf; LW, the maximum horizontal width of the leaf; and SW, calculated as the weight of 1000 seeds.

For qualitative traits, FC was recorded after flowering, and colour changes during development were indicated with a transition suffix “_T” (e.g., YE_T for a transition from yellow). FC was categorized as YE, WT, or RD. SCC was classified as WT, BN, DB, LB, or MX. The presence and size of TI were evaluated and classified as NT, LT, or ST. LM was assessed for structural characteristics and categorized as M, N, or P.

### 4.4. Seed Crude Extract Preparation

Seed crude extracts were prepared following an established method [15]. Briefly, 7 g of finely ground safflower seed powder was mixed with 20 mL of 75% ethanol and extracted using an accelerated solvent extractor (ASE-350; Dionex, Sunnyvale, CA, USA) under nitrogen gas. Extraction conditions were set to 1200 psi and 70 °C. The resulting extracts were concentrated in a vacuum concentrator (HT-6; Genevac, Ipswich, UK) at 40 °C for 10 h. The concentrated samples were reconstituted in 75% ethanol, filtered through a 0.45 µm membrane, and adjusted to final concentrations of 0.1 mg/mL, 0.2 mg/mL, and 5 mg/mL for TPC, antioxidant activity, and serotonin derivative analyses, respectively.

### 4.5. Determination of TPC

TPC was determined using the Folin–Ciocalteu colorimetric assay, as described by Waterhouse [16] and modified by others [17]. Briefly, 100 µL of sample or standard was mixed with 100 µL of Folin–Ciocalteu reagent and incubated at 23–25 °C for 3 min. Then, 100 µL of 2% Na_2_CO_3_ was added, and the mixture was incubated in the dark at 23–25 °C for 30 min. Absorbance was measured at 750 nm using an Eon Microplate Spectrophotometer (Bio-Tek, Inc., Winooski, VT, USA). TPC was quantified using a standard calibration curve prepared with gallic acid and expressed as gallic acid equivalent (µg·GAE/mg).

### 4.6. Analysis of Serotonin Derivative Contents

The levels of CS and FS were quantified using an Agilent 1290 ultra-performance liquid chromatography (UPLC) system (Agilent Technologies, Santa Clara, CA, USA) [18]. The mobile phase consisted of solvent A (0.1% formic acid in water) and solvent B (0.1% formic acid in acetonitrile). A gradient elution program was applied as follows: 0–6 min, 85% A and 15% B; 6–15 min, 85–60% A and 15–40% B; and 15–20 min, 60–20% A and 40–80% B. Detection was performed at 324 nm using an Eclipse Plus C18 column (1.8 μm, 2.1 × 50 mm). The injection volume, flow rate, and column temperature were set to 2 µL, 0.4 mL/min, and 25 °C, respectively. CS and FS were identified based on their retention times (3.65 and 3.88 min, respectively) and quantified using standard calibration curves: y = 12.402x − 9.8267 (R^2^ = 0.9999) for CS and y = 24.485x − 93.577 (R^2^ = 0.9995) for FS.

### 4.7. Antioxidant Capacity Assay

Antioxidant activities were evaluated using DPPH and ABTS assays, following modified protocols [19,20]. For the DPPH assay, 100 µL of purified crude extract was mixed with 150 µL of 150 µM DPPH solution, followed by incubation in the dark at 25 °C for 30 min. Absorbance was measured at 517 nm. For the ABTS assay, ABTS stock solution (7 mM) was mixed with potassium persulfate (2.45 mM) and incubated overnight in the dark at 23–25 °C. The solution was then diluted with methanol to an absorbance of 0.7 ± 0.02 at 734 nm. Subsequently, 10 µL of sample was added to 190 µL of the ABTS radical solution, and absorbance was measured at 734 nm after 6 min. Antioxidant activities were calculated using the following formula:

Antioxidant activity (%) = [1 − (A_sample_ − A_sample blank_)/(A_control_ − A_control blank_)] × 100

### 4.8. Determination of Total Oil Content

Total oil content was analyzed using a Soxhlet apparatus (Soxtec™ 2043 system; OSS Tecator AB, Hillerod, Denmark). One gram of seed powder was mixed with 50 mL of *n*-hexane and subjected to extraction at 135 °C in three stages: boiling (30 min), rinsing (60 min), and recovery (30 min). The extracted oil was weighed, and oil content was expressed as a percentage of the seed sample weight.

### 4.9. Analysis of Fatty Acids

Fatty acid profiles were analyzed after derivatization to fatty acid methyl esters. Each sample tube containing 50 μL of crude fat was mixed with 2 mL of NaOH, vortexed for 5 s, heated at 80 °C for 10 min, and cooled to room temperature for 5 min. Then, 2 mL of 14% BF_3_–methanol was added, vortexed for 5 s, reheated at 80 °C for 10 min, and cooled again for 5 min. Subsequently, 7 mL of *n*-hexane and 2 mL of H_2_O were added, vortexed for 10 s, and centrifuged at 3600 rpm at 4 °C for 10 min. The supernatant (*n*-hexane layer) was collected, filtered through anhydrous sodium sulfate, transferred to sample vials, and stored at −20 °C for gas chromatography analysis.

Analysis was performed using a GCMS-QP2010 Ultra Gas Chromatography instrument (Shimadzu Co., Kyoto, Japan) equipped with an HP-INNOWAX column (0.25 mm × 30 m, 0.25 μm; Agilent Technologies Inc., Santa Clara, CA, USA). The column temperature was initially set at 100 °C, increased to 170 °C at 60 °C/min, held for 1 min, and then further increased to 240 °C at the same rate, where it was held for an additional 1 min. The injector and detector temperatures were maintained at 250 °C. A 1 µL sample was injected with a split ratio of 50:1, and helium was used as the carrier gas at a flow rate of 1.5 mL/min and a pressure of 117 kPa. Individual fatty acids were identified by comparing their retention times (RT) with those of known standards. Fatty acid contents were calculated and expressed as percentages of total fatty acids based on peak areas.

### 4.10. Statistical Analysis

All experiments were performed in triplicate, and results are presented as mean ± standard deviation. Statistical analyses, including ANOVA, Pearson correlation, PCA, and hierarchical clustering analysis (HCA), were conducted using R software (version 4.2.2; RStudio, Boston, MA, USA).

## 5. Conclusions

This study underscores the central role of FC in shaping phenotypic diversity in safflower, influencing both quantitative and qualitative traits. FC exhibited significant associations with key traits such as OA, LA, ODR, and FS, demonstrating its impact on metabolic and agronomic characteristics. PCA and network analyses revealed complex trait interactions and distinct phenotypic patterns based on FC, providing insights into the underlying relationships. Furthermore, strong correlations between FC, SCC, and TI highlight the potential of FC as a phenotypic marker for breeding programs. By integrating advanced statistical and network-based approaches, this study offers a comprehensive framework for enhancing safflower breeding efforts, with a focus on oil quality, antioxidant properties, and morphological traits. Future research should prioritize genetic and molecular studies to validate these findings and explore their application in the development of high-performing safflower varieties.

## Figures and Tables

**Table 1:** Abbreviations used in this study.

Abbreviation	Full Term
FC	Flower color
SCC	Seed coat color
FD	Flowering date (days)
MD	Maturing date (days)
LL	Leaf length (cm)
LW	Leaf width (cm)
SW	1000-seed weight (g)
TI	Thorn of involucre
TO	Total oil (%)
PA	Palmitic acid (%)
SA	Stearic acid (%)
OA	Oleic acid (%)
LA	Linoleic acid (%)
ALA	Alpha-linolenic acid (%)
TSFA	Total saturated fatty acid (%)
TUFA	Total unsaturated fatty acid (%)
ODR	Oleic de-saturation ratio (%)
LDR	Linoleic de-saturation ratio (%)
TPC	Total phenolic content (µg·GAE/mg·DE)
ABTS	2,2′-azino-bis (3-ethylbenzothiazoline-6-sulfonic acid) (%)
DPPH	2,2-diphenyl-1-picrylhydrazyl (%)
CS	*N*-(*p*-coumaroyl)serotonin (%)
FS	*N*-feruloylserotonin (%)

**Table 2:** Analysis of eta-squared effect sizes and *p*-values for quantitative traits associated with flower color (FC) in safflower.

Parameter	Trait ^z^	Eta Squared (η^2^)	Effect Size Interpretation	*p*-Value
FC	FD	0.06	Medium	0.016 *
	MD	0.04	Small	0.061
	LL	0.02	Small	0.331
	LW	0.05	Small	0.038 *
	SW	0.01	Small	0.509
	TO	0.04	Small	0.066
	PA	0.07	Medium	0.009 **
	SA	0.01	Small	0.483
	OA	0.22	Large	0.000 **
	LA	0.22	Large	0.000 **
	ALA	0.04	Small	0.084
	TSFA	0.03	Small	0.137
	TUFA	0.03	Small	0.138
	ODR	0.22	Large	0.000 **
	LDR	0.04	Small	0.068
	TPC	<0.01	Negligible	0.779
	ABTS	0.11	Medium	0.000 **
	DPPH	0.03	Small	0.145
	CS	0.03	Small	0.156
	FS	0.17	Large	0.000 **
SCC	FD	0.03	Small	0.227
	MD	0.03	Small	0.204
	LL	0.03	Small	0.325
	LW	<0.01	Negligible	0.905
	SW	0.04	Small	0.115
	TO	0.18	Large	0.000 **
	PA	0.16	Large	0.000 **
	SA	0.01	Small	0.675
	OA	0.02	Small	0.350
	LA	0.03	Small	0.317
	ALA	0.02	Small	0.516
	TSFA	0.05	Small	0.063
	TUFA	0.05	Small	0.063
	ODR	0.03	Small	0.345
	LDR	0.02	Small	0.453
	TPC	0.04	Small	0.133
	ABTS	0.24	Large	0.000 **
	DPPH	0.08	Medium	0.012 *
	CS	0.09	Medium	0.005 **
	FS	0.11	Medium	0.001 **

^z^ FD: flowering date (days). MD: maturing date (days). LL: leaf length (cm). LW: leaf width (cm). SW: 1000-seed weight (g). TO: total oil (%). PA: palmitic acid (%). SA: stearic acid (%). OA: oleic acid (%). LA: linoleic acid (%). ALA: alpha-linolenic acid (%). TSFA: total saturated fatty acid (%). TUFA: total unsaturated fatty acid (%). ODR: oleic de-saturation ratio (%). LDR: linoleic de-saturation ratio (%). TPC: total phenolic content (µg·GAE/mg·DE). ABTS: 2,2′-azino-bis(3-ethylbenzothiazoline-6-sulfonic acid) (%). DPPH: 2,2-diphenyl-1-picrylhydrazyl (%). CS: *N*-(*p*-coumaroyl)serotonin (%). FS: *N*-feruloylserotonin. (%). Statistical significance levels: * *p* < 0.05, ** *p* < 0.01.

**Table 3:** Chi-square analysis of safflower traits: Association strengths based on *p*-values and Cramer’s V.

Trait ^z^	Chi-Square (X^2^)	df	*p*-Value	Cramer’s V	Interpretation
SCC	28.606	12	0.005 **	0.2354541	Moderate
TI	23.234	6	0.001 **	0.259888	Moderately Strong
LM	6.7822	6	0.342	0.1404129	Very Weak

^z^ SCC: seed coat color. TI: thorn of involucre. LM: leaf margin. Statistical significance levels: ** *p* < 0.01.

## Data Availability

Data will be made available on request.

## Supplementary Materials

The following supporting information can be downloaded at: https://www.mdpi.com/article/10.3390/ijms26073105/s1.
