# Supplementary material for: Flower Color and Seed Coat Color as a Phenotypic Marker: Correlations with Fatty Acid Composition, Antioxidant Properties, and Metabolite Profiles in Safflower (Carthamus tinctorius L.)
Source: Int J Mol Sci. 2025 Mar 27;26(7):3105. doi: 10.3390/ijms26073105 (PMC11988472; doi:10.3390/ijms26073105)
Supplement: Supplementary file 1 [file ijms-26-03105-s001.zip › ijms-3450080-supplementary.pdf]

Supplementary Materials

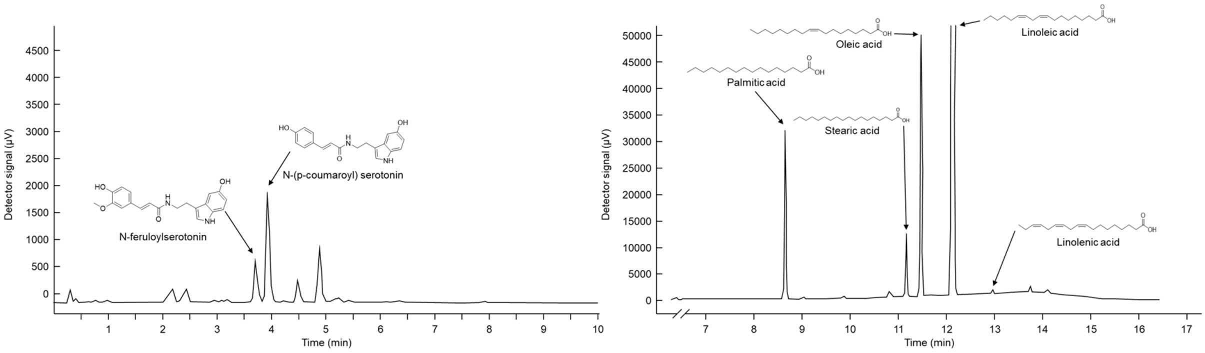

Figure S1. Gas chromatography (GC) chromatograms illustrating the detection of serotonin derivatives (*N*-feruloylserotonin and *N*-(*p*-coumaroyl)serotonin) and fatty acid components (palmitic acid, stearic acid, oleic acid, linoleic acid, and linolenic acid). The retention time (x-axis) and detector response (y-axis) are displayed, with labeled peaks corresponding to specific compounds identified in the sample. These chromatograms offer a comprehensive visualization of the metabolic profile and fatty acid composition analyzed in this study.

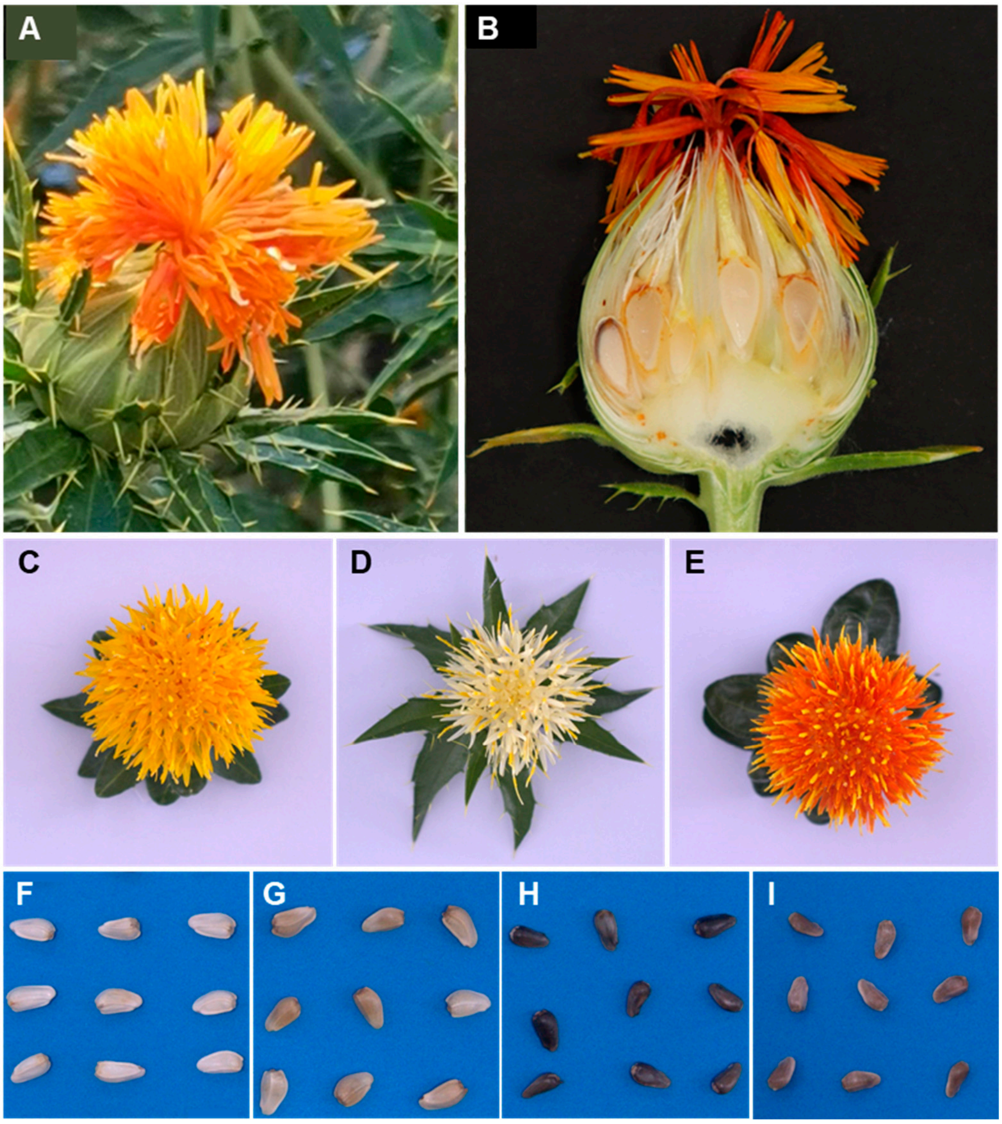

Figure S2. Morphological variation in flower and seed coat color among safflower accessions. **(A)** Side view of the safflower capitulum, showing the arrangement and coloration of the florets. **(B)** Longitudinal section of a safflower capitulum, highlighting the internal floral structure and developing seeds. **(C–E)** Variation in flower color among accessions: yellow **(C)**, white **(D)**, and red **(E)**. **(F–I)** Variation in seed coat color among accessions: white **(F)**, light brown **(G)**, dark brown **(H)**, and brown **(I)**. One-to-one mapping between flower and seed images was not applied due to the presence of seed coat color variation within each flower color category.

Table S1. Description of safflower accessions used in the study.

No.	IT No.	Accession name	Origin
1	K184954	PI 253388	Spain
2	IT345396	PI 252042	Turkey
3	K184960	PI 253394	Spain
4	K184877	PI 250712	Iran
5	K185035	PI 253911	Afghanistan
6	K185034	PI 253910	Afghanistan
7	IT345394	PI 251989	Turkey
8	IT345397	PI 253549	Portugal
9	K184880	PI 250715	Iran
10	K184836	PI 250481	Pakistan
11	K185009	PI 253570	Portugal
12	IT345401	PI 253555	Portugal
13	IT345403	PI 253559	Portugal
14	IT345385	PI 250599	Pakistan
15	K184882	PI 250717	Iran
16	IT345395	PI 252040	Turkey
17	IT345392	PI 251986	Turkey
18	IT345400	PI 253554	Portugal
19	K185003	PI 253563	Portugal
20	IT345386	PI 250819	Iran
21	K184881	PI 250716	Iran
22	IT345393	PI 251987	Turkey
23	K184945	PI 251988	Turkey
24	IT345377	PI 250197	Pakistan
25	K184805	PI 250198	Pakistan
26	IT345376	PI 250196	Pakistan
27	IT345380	PI 250343	Pakistan
28	K185074	PI 259996	Pakistan
29	K185261	PI 304453	Iran
30	K185266	PI 304458	Iran
31	K185134	PI 262453	China
32	K185259	PI 304451	Iran
33	IT345405	PI 301048	Turkey
34	IT345384	PI 250598	Pakistan
35	K185273	PI 304465	Iran
36	IT345399	PI 253553	Portugal

37	K185067	PI 259989	Pakistan
38	K185070	PI 259992	Pakistan
39	K184911	PI 250840	Iran
40	K184891	PI 250820	Iran
41	IT345381	PI 250353	Pakistan
42	IT345378	PI 250204	Pakistan
43	K185267	PI 304459	Iran
44	K185045	PI 254717	Iran
45	K185048	PI 255577	Iran
46	K185052	PI 257582	Ethiopia
47	IT345404	PI 254363	India
48	K185069	PI 259991	Pakistan
49	K185136	PI 268374	Afghanistan
50	IT345379	PI 250208	Pakistan
51	K184833	PI 250478	Pakistan
52	IT345382	PI 250474	Pakistan
53	IT345383	PI 250475	Pakistan
54	K185051	PI 257291	Iran
55	K184909	PI 250838	Iran
56	IT345389	PI 251981	Turkey
57	K185043	PI 254365	India
58	K185042	PI 254364	India
59	IT345388	PI 250920	Iran
60	IT345390	PI 251982	Turkey
61	IT345391	PI 251984	Turkey
62	K185268	PI 304460	Iran
63	IT345413	Kransovodopadskij 4	Kazakhstan
64	K185750	PI 343773	Iran
65	K185780	PI 380800	Iran
66	K185792	PI 388907	Iran
67	K185794	PI 388909	Iran
68	IT345420	PI 451957	India
69	K185944	Huaxian	China
70	K185991	W6 3568	United States of America
71	IT345421	Xichang	China
72	K185976	W6 3553	United States of America
73	K185527	PI 306944	India
74	K185529	PI 306946	India
75	K185725	PI 340075	Turkey

76	IT345407	PI 305187	India
77	K185531	PI 306948	India
78	IT345415	PI 401585	India
79	IT345416	PI 401588	India
80	IT345417	PI 406003	Iran
81	K185789	PI 388904	Iran
82	K185759	PI 343782	Iran
83	K185751	PI 343774	Iran
84	IT345414	PI 388903	Iran
85	IT345412	PI 343930	Ethiopia
86	IT345411	PI 343781	Iran
87	IT345409	PI 340095	Turkey
88	K185749	PI 343772	Iran
89	K185728	PI 340078	Turkey
90	K185727	PI 340077	Turkey
91	K185726	PI 340076	Turkey
92	IT345418	PI 407606	Turkey
93	IT345408	PI 340094	Turkey
94	IT345410	PI 340096	Turkey
95	K185738	PI 340088	Turkey
96	K185733	PI 340083	Turkey
97	K185735	PI 340085	Turkey
98	K185279	PI 304471	Iran
99	K185284	PI 304476	Iran
100	K184906	PI 250835	Iran
101	K184898	PI 250827	Iran
102	K184896	PI 250825	Iran
103	K184897	PI 250826	Iran
104	K184900	PI 250829	Iran
105	K185986	W6 3563	United States of America
106	K185346	PI 305191	India
107	IT345419	PI 407615	Turkey
108	K185345	PI 305190	India
109	K185277	PI 304469	Iran
110	K185337	PI 305179	India
111	K185723	PI 340073	Turkey
112	K185278	PI 304470	Iran
113	K185276	PI 304468	Iran
114	K185283	PI 304475	Iran

115	K184904	PI 250833	Iran
116	K185274	PI 304466	Iran
117	K185333	PI 305174	India
118	K184903	PI 250832	Iran
119	K184901	PI 250830	Iran
120	K184905	PI 250834	Iran
121	IT345406	PI 305175	India
122	K185275	PI 304467	Iran
123	K186344	BULK 731	United States of America
124	IT345428	BULK 734A	United States of America
125	K186420	PI 613371	United States of America
126	K186489	PI 613441	United States of America
127	K186192	23-2	United States of America
128	K186206	Demo 138	United States of America
129	K186076	W6 3655	United States of America
130	K186122	PI 544018	China
131	K186475	PI 613427	United States of America
132	K186466	PI 613417	United States of America
133	K186455	PI 613406	United States of America
134	K186486	PI 613438	United States of America
135	K186347	BULK 735	United States of America
136	K186422	PI 613373	United States of America
137	K186160	PI 544056	China
138	K186424	PI 613375	United States of America
139	K186342	BULK 727 732	United States of America
140	K186334	Ariz Safflower Composite I	United States of America
141	K186362	B 8-5-5	United States of America
142	K186073	W6 3652	United States of America
143	K186065	W6 3644	United States of America
144	K186063	W6 3642	United States of America
145	K186055	W6 3634	United States of America
146	K186439	PI 613390	United States of America
147	K186432	PI 613383	United States of America
148	K186043	W6 3621	United States of America
149	K186048	W6 3627	United States of America
150	K186023	W6 3600	United States of America
151	IT345429	PI 613389	United States of America
152	K186442	PI 613393	United States of America
153	K186446	PI 613397	United States of America

154	K186431	PI 613382	United States of America
155	K186427	PI 613378	United States of America
156	K186207	Demo 149	United States of America
157	K186239	PI 568801	China
158	K186186	11-2	United States of America
159	K186053	W6 3632	United States of America
160	IT345427	W6 3633	United States of America
161	K186074	W6 3653	United States of America
162	IT345423	W6 3575	United States of America
163	IT345424	W6 3623	United States of America
164	IT345422	W6 3573	United States of America
165	K186452	PI 613403	United States of America
166	K186476	PI 613428	United States of America
167	K186009	W6 3586	United States of America
168	K186024	W6 3601	United States of America
169	K186067	W6 3646	United States of America
170	K186075	W6 3654	United States of America
171	K186070	W6 3649	United States of America
172	IT345425	W6 3624	United States of America
